# 117 Implementation Methods, Experiences, and Perceptions of Enhanced Barrier Precautions in Florida Nursing Homes

**DOI:** 10.1017/ash.2026.10720

**Published:** 2026-06-23

**Authors:** Shalini Gore, Eleanor McQuillen, Chitra Pai, Jackie Chan

**Affiliations:** 1 Touro University California College of Osteopathic Medicine; 2 Touro University College of Osteopathic Medicine; 3 Touro University California

## Abstract

**Background:** Antimicrobial resistance (AMR) among urinary pathogens threatens the effective treatment of urinary tract infections (UTIs) worldwide, but regional surveillance data from Western India remains limited. In this study, we aimed to characterize trends of the most prevalent uropathogens from multiple medical centers in Maharashtra and identify patterns within their AMR profiles. **Methods:** This is a multicentric international collaborative project using de-identified patient samples collected from multiple healthcare sites in Navi Mumbai. 300 urine samples were collected by our collaborative medical laboratory and processed in accordance with the Clinical and Laboratory Standards Institute (CLSI) guidelines. Across gender and age groups, data was analyzed for the distribution of uropathogens and their AMR patterns. **Results:** A total of 129 (43%) positive urine cultures were identified including 90 samples from women (69.8%) and 39 from men (30.2%). Gram-negative bacteria were isolated from 116 (89.9%) positive cultures, with a predominance of Escherichia coli (46.6%), Klebsiella pneumoniae (37.9%), and Acinetobacter baumannii (11.2%). E. coli was the most prevalent isolate in ages 19-50 (52%) followed by K. pneumoniae (34.7%). In ages 51-90, K. pneumoniae predominated (43.9%) followed by E. coli (36.6%). Prevalence of A. baumannii was modestly higher than reports from neighboring regions in India. 68.5% of E. coli and 50% of K. pneumoniae isolates showed multi-drug resistance (MDR). E. coli showed the highest resistance to penicillins (94.4%), oral cephalosporins (66.7%), and beta-lactam/beta-lactamase inhibitors (53.7%). K. pneumoniae was most resistant to penicillins (100%), beta-lactam/beta-lactamase inhibitors (91.3%), and fluoroquinolones (50%). Resistance to fluoroquinolones (50%) in both species was lower than reported in previous studies from Western India (70–78%). The highest sensitivities for E. coli and K. pneumoniae were observed with carbapenems (100%, 97.7%), nitrofurantoin (94.4%, 88.6%), and amikacin (94.4%, 86.4%). **Conclusion:** Our study emphasizes the need for ongoing monitoring of predominant uropathogens and their AMR profiles at the regional level. Demographic differences between the prevalence of E. coli vs K. pneumoniae suggests potential host or exposure factors influencing pathogen distribution, warranting further research. The strong activity of nitrofurantoin supports its continued use as first-line empiric therapy.

